# Research on Phthalic Acid Esters Removal and Its Health Risk Evaluation by Combined Process for Secondary Effluent of Wastewater Treatment Plant

**DOI:** 10.1155/2013/952780

**Published:** 2013-07-11

**Authors:** Simin Li, Yongkang Lv, Na Zhao

**Affiliations:** ^1^Key Laboratory of Coal Science and Technology, Ministry of Education and Shanxi Province, Taiyuan University of Technology, Taiyuan 030024, China; ^2^Hebei University of Engineering, Handan 056038, China

## Abstract

This paper analyses the treatment effect of the “coagulation-sedimentation-O_3_-biological sand filtration-GAC” combined process on phthalic acid esters in secondary effluent of municipal wastewater treatment plant and meanwhile evaluate its health risk. The results indicated that when the concentrations of DBP and DiOP in secondary effluent were at range of 0.41 mg/L–0.814 mg/L and 0.23 mg/L–0.36 mg/L, the average total removal rates of DBP and DiOP were 85.10% and 68.11%, and the average concentration of DBP and DiOP in effluent were 0.089 mg/L and 0.091 mg/L, respectively. The quality of the effluent met the requirement of the ornamental scenic environment water in *The Quality of Urban Wastewater Recycling and Scenic Environment Water (GB/T 18921-2002)*, and the health risks of DBP and DiOP in effluent were at range of 1.99 × 10^−12^ –2.15 × 10^−12^/a and 1.48 × 10^−11^ –1.85 × 10^−11^/a, respectively, which is lower than the acceptable maximum risk level: 1.0 × 10^−6^.

## 1. Introduction

With the development of reclaimed water returning to agricultural irrigation, industrial cooling, urban landscape, and so forth, people increasingly are concerned about PAEs' impact on human health [1]. Six compounds in PAEs have been listed by USEPA as precedence-controlled pollutants three compounds have been listed by environmental protection bureau of our country as environmental priority pollutants [2, 3]. At present, PAEs have also been detected in ecosystem of many industrial countries around the world [4]. PAEs are a kind of endocrine disrupting chemicals which exists in environment; it is toxic and can enter into human body, strengthen the possibility of damaging the human chromosome, and block the normal growth of human and animal and regeneration of white blood cells of human [5, 6]. It can cause cancer, teratogenesis, and mutagenicity [7, 8]. For this reason, it is vital to research on the removal of phthalic acid esters in reclaimed water and analyse its health risk. This paper studies adsorption and removal effect of the “coagulation-sedimentation-O_3_-biological sand filtration-GAC” combined process on phthalic acid esters in secondary effluent from municipal wastewater treatment plant and evaluates the risk of effluent returning to landscape water on human health.

## 2. Raw Water Quality and Methods

### 2.1. Quality of the Raw Water

The raw water came from secondary effluent of a wastewater treatment plant, and conventional water quality indexes during the test are shown in Table 1.

### 2.2. Experimental Process

**Figure 1:**
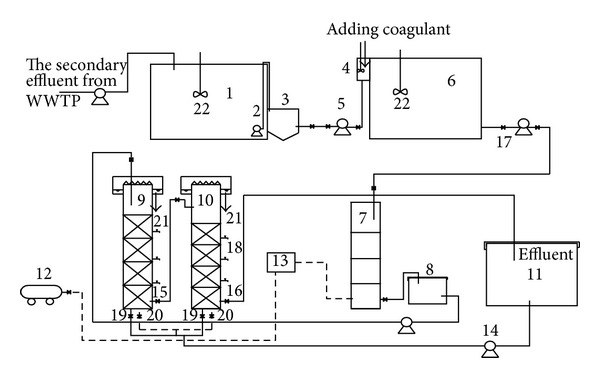
Process flow chart: (1) raw water tank; (2) submersible sewage pump; (3) grit chamber; (4) mixing tank; (5) peristaltic pump; (6) coagulative precipitation tank; (7) ozonation contact column; (8) ozone release tank; (9) sand filter column; (10) active carbon; (11) solution tank; (12) air compressor; (13) ozone generator; (14) backwash return pump; (15) sand filter column water outlet; (16) active carbon column water outlet; (17) magnetic valve; (18) sample tap; (19) back wash water inlet; (20) back wash water inlet; (21) back wash air inlet; (22) stirrer.

The process flow is shown in Figure 1. Secondary effluent was pumped by pump to raw water tank, it flowed into inclined tube settler after mixed with chemicals and then flowed into ozone contact column, and finally the effluent flowed into sand filter column and active carbon column. The regular running parameters of the experiment are as follows: optimal ozone dosage: 3 mg/L; hydraulic loading of sand filter column: 6 m^3^/m^2^·h; hydraulic loading of active carbon column: 5 m^3^/m^2^·h.

### 2.3. Experimental Process

The process flow is shown in Figure 1. Secondary effluent was pumped by submersible sewage pump to raw water tank, it flowed into inclined tube settler after being mixed with chemicals and then flowed into ozone contact column, and finally the effluent of ozone contact column flowed into sand filter column and active carbon column. The regular running parameters of the experiment are as follows: optimal ozone dosage: 3 mg/L; hydraulic loading of sand filter column: 6 m^3^/m^2^·h; hydraulic loading of active carbon column: 5 m^3^/m^2^·h.

### 2.4. Experiment Methods

The water turbidity was determined by turbidity meter (2100P) of HACH. Chromaticity (UV_400_) and ultraviolet absorbency degree (UV_254_) were determined by ultraviolet spectrophotometer (domestic UV1102). COD_Mn_ was determined by potassium permanganate method [2].

Through analyzing the type and concentration of PAEs, two typical types of PAEs were studied: DBP (C_16_H_22_O_4_, molecular weight 278.15) and DiOP (C_24_H_38_O_4_, molecular weight 390.28), and gas chromatograph (HP6890/5973) from Agilent Technologies (USA) was used in this experiment. The health risk was measured by the reference dose of the compound exposure.

## 3. Analysis on the Removal of PAEs by Combined Process

**Figure 2:**
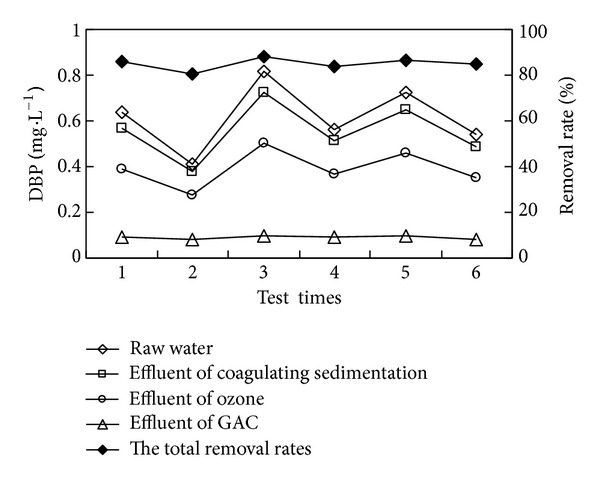
The removal effect of DBP in water by combined process.

**Figure 3:**
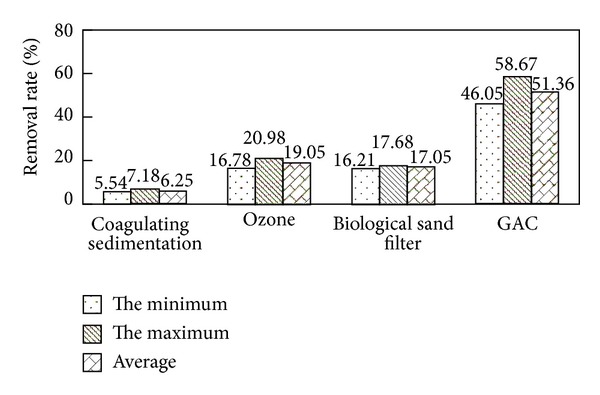
The removal effect of DBP in water by each processing unit of combined process.

**Figure 4:**
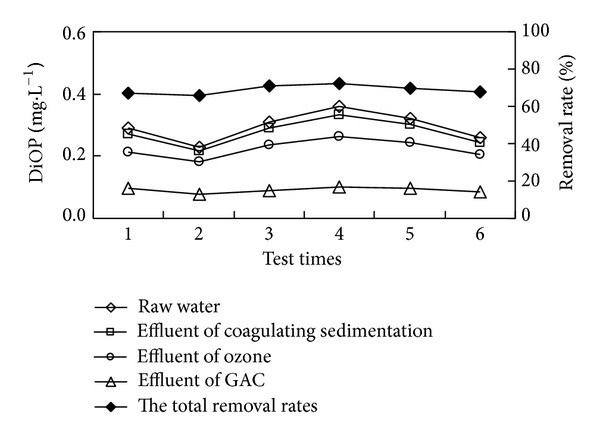
The removal effect of DiOP in water by combined process.

**Figure 5:**
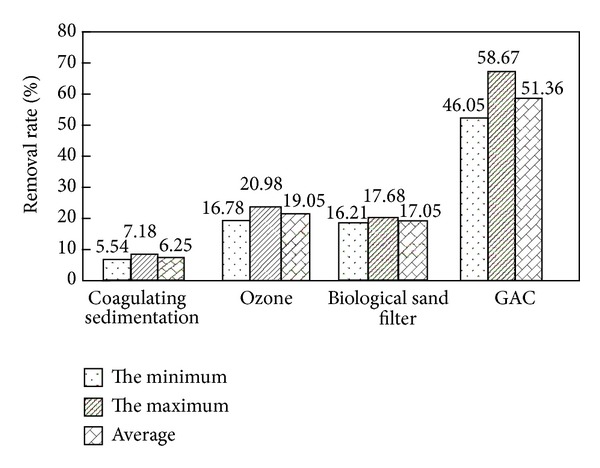
The removal effect of DiOP in water by each processing unit of combined process.

“Coagulation-sedimentation-O_3_-biological sand filter-GAC” combined process was adopted to degrade DBP, DiOP in secondary effluent. The concentration and removal rate of DBP and DiOP in each unit of the combined process were shown in Figures 2 to 5.

As shown in Figures 2 and 4, when DBP and DiOP in secondary effluent were at range of 0.41 mg/L–0.814 mg/L and 0.23 mg/L–0.36 mg/L, respectively, the removal rates of DBP and DiOP by combined process were at range of 80.79%–88.18% and 65.54%–72.15%, respectively. Average total removal rates were 85.10% and 68.81%, respectively, and the concentrations of DBP and DiOP in effluent of the process were at range of 0.078 mg/L–0.099 mg/L and 0.079 mg/L–0.100 mg/L, respectively, while average concentrations were 0.089 mg/L and 0.091 mg/L, respectively. From Figures 3 and 5, the removal rates of DBP and DiOP in raw water of coagulating sedimentation unit were at range of 8.08%–10.94% and 5.54%–7.18%, respectively, and average removal rates were 9.92% and 6.25%, respectively. The removal rates of DBP and DiOP in effluent of coagulating sedimentation by ozone contact unit were at range of 26.33%–31.58% and 16.78%–20.98%, respectively, and average removal rates were 28.99% and 19.05%, respectively; the removal rates of DBP and DiOP in effluent of ozone contact column by biological sand filter column were at range of 20.01%–23.51% and 16.21%–17.68%, respectively, and average removal rates were 22.01% and 17.05%, respectively. The removal rates of DBP and DiOP in effluent of biological sand filter column by active carbon column unit were at range of 64.53%–74.89% and 46.05%–58.67%, respectively, and average removal rates were 70.28% and 51.36%, respectively. From the total removal rates in Figures 3 and 5, we can see that the removal rate of DBP by combined process is larger than the removal rate of DiOP by combined process due to the reason of molecular weight of DBP being lower than that of DiOP, and small molecular substances are more easily to be oxidated and absorbed by ozone unit and active carbon unit [4]. By the experimental analysis from Figures 2 to 5, we can see that the removal of trace organic substance sedimentation works by flocculating constituent; it can be removed by the sedimentation of flocculating constituent which is by the formation of flocculation of suspended particle and colloid in water, and this is mainly because PAEs are a kind of hydrophobic organic compounds which show a strong affinity with the surface of inorganic mineral [10]. Strong oxidizing property of ozone can change the structure of trace organic substances and disconnect their chemical bonds, and DBP and DiOP can be degraded to phthalic acid lipid, phthalic acid, or even low molecular weight organic matter such as aldehyde, ketone, and acid, and meanwhile strengthen the degradation property of the following biological sand filter and the absorption property of the active carbon. Biological sand filter column unit works by the degradation property of the microorganism and the physical entrapment property of the sand filter; meanwhile it reduces the burden of the following active carbon column and extends the lifespan. Active carbon can eradicate the organic matters where the molecular size as well as polarity after oxidation is similar to the physical property; active carbon has a certain effect on the removal of trace organic substances.

## 4. Evaluation on Health Risk of PAEs

### 4.1. Computing Method of Health Risk

According to the Integrated Risk Information System (IRIS) from USEPA, DBP and DiOP are noncarcinogens, and their exposure reference doses are 1.00 × 10^−1^ mg/(kg·d), and 2.00 × 10^−2^ mg/(kg·d) respectively. The mathematical model of health risk evaluation on sole non carcinogens chemicals is as follows [11]:

(1) P=D(RfD×70)×10−6,

where *P* is the individual health risk when certain health risk happens, dimensionless; *D* is exposure dose per day on body weight unit of non carcinogens pollutants, mg/(kg·d); RfD is reference dose of certain chemical substance with threshold, mg/(kg·d).

Analytical calculations of PAEs' health risks are shown in Table 2.

**Figure 6:**
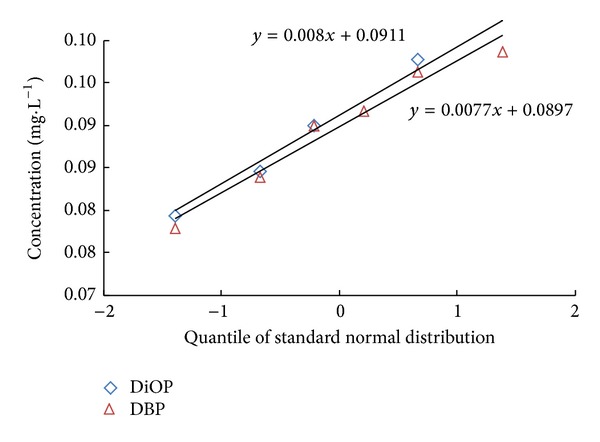
Q-Q graph of DBP and DiOP in effluent of combined process.

The dose and reaction relations of the trace pollutants need to be determined before this calculation method was applied, by using Q-Q graphs which satisfy Gaussian distribution to determine whether DBP and DiOP satisfy Gaussian distribution. Q-Q graph of DBP and DiOP in combined process is shown in Figure 6.

From Figure 6, it can be seen that Q-Q graph of DBP and DiOP shows a straight line which means that two substances follow Gaussian distribution.

### 4.2. Health Risk of DBP and DiOP

According to health risk computing model, the risks of DBP and DiOP are shown in Table 3. 

From Table 3, it can be seen that the health risks of DBP and DiOP on human were at range of 1.99 × 10^−12^–2.15 × 10^−12^/a and 1.02 × 10^−11^–1.11 × 10^−11^/a, respectively, which is lower than internationally recognized ignorable level, so the health risk of DBP in effluent can be ignored [11]. Then it can be concluded that the “coagulation-sedimentation-O_3_-biological sand filter-GAC” combined process is feasible to treat the secondary effluent.

## 5. Conclusion

The recycling of municipal wastewater treatment plant's secondary effluent is a better way to alleviate the shortage of water resources. It can save the limited fresh water resources effectively. A combined process of “coagulation-sedimentation-O_3_-biological sand filtration-GAC” was adopted to treat the secondary effluent, and the removal of phthalic acid esters and its health risk evaluation by combined process were investigated.

1. The average removal rate of DBP in influent by coagulation sedimentation units was 9.92%; the average removal rate of DBP in effluent of coagulating sedimentation by ozone column unit was 28.99%; the average removal rate of DBP in effluent of ozone contact column by biological sand filter column was 22.01%; the average removal rate of DBP in effluent of biological sand filter by active carbon unit was 70.28%.
2. The average removal rate of DiOP in influent of the process by coagulating sedimentation unit was 6.25%; the average removal rate of DiOP in effluent of coagulating sedimentation by ozone contact column unit was 19.05%; the average removal rate of DiOP in effluent of ozone by biological sand filter column was 17.05%; the average removal rate of DiOP in effluent of biological sand filter column by active carbon column was 51.36%.
3. The removal rate of DBP by combined process was higher than that of the removal rate of DiOP due to the high molecular weight of DiOP, and small molecular substances were more easily oxidized and absorbed by ozone contact column unit the organic matter with small molecular weight can be easily degraded by sand filter.
4. The concentration of DBP and DiOP in effluent of the combined process follows Gaussian distribution (Q-Q graph shows a straight line), and the health risk on human was at range of 1.99 × 10^−12^–2.15 × 10^−12^/a and 1.02 × 10^−11^–1.11 × 10^−11^/a, respectively.
5. The average removal rates of DBP and DiOP in secondary effluent by “coagulation-sedimentation-O3-biological sand filter-GAC” combined process were 85.10% and 68.81%, respectively, the average removal rates of DBP and DiOP in effluent were 0.089 mg/L and 0.091 mg/L, respectively, and the quality of effluent met the standard of *The Quality of Urban Wastewater Recycling and Scenic Environment Water (GB/T 18921-2002)*; the health risk of DBP and DiOP in effluent was lower than the maximum human acceptable risk level: 1.0 × 10^−6^.

## Figures and Tables

**Table 1:** The quality of raw water.

Water quality index	Water temperature (°C)	pH	Turbidity (NTU)	COD_Mn_ (mg/L)	Chroma (degree)	UV_254_ (cm^−1^)
Variation range	20–28	7-8	1–10 (5.6)	5–30 (13.4)	10–50 (29.7)	0.1–0.25 (0.164)

Numbers in ( ) are average value.

**Table 2:** PAEs' health risk calculating table.

Calculating parameters	Values	Note
Concentration of the pollutants *C* (mg/L)	(*μ*, *σ*)	Follow Gaussian distributions
Reference dose mg/(kg·d)	RfD	By mouth
Exposure volume at one time *V* (mL)	100	
Exposure times per year (*n*)	40	
Average body weight (kg)	70	
Average lifespan (a)	70	
Average exposure dose per unit of body mass	*d* = (*n* × *V* × 10^−3^ × *C*)/365 × 70	
Lifelong health risk *P*	*P* = *d* × 10^−6^/RfD	
Individual health risk Pa	Pa = *P*/70	
Maximum acceptable risks [9]	1.0 × 10^−6^	

**Table 3:** The quantile table of the life risk and the annual risk of DiOP.

Quantile	DBP		DiOP	
	*P*	Pa	*P*	Pa
0%	1.39*E* − 10	1.99*E* − 12	7.12*E* − 10	1.02*E* − 11
10%	1.40*E* − 10	2.01*E* − 12	7.19*E* − 10	1.03*E* − 11
20%	1.42*E* − 10	2.02*E* − 12	7.25*E* − 10	1.04*E* − 11
30%	1.43*E* − 10	2.04*E* − 12	7.31*E* − 10	1.04*E* − 11
40%	1.44*E* − 10	2.05*E* − 12	7.37*E* − 10	1.05*E* − 11
50%	1.45*E* − 10	2.07*E* − 12	7.44*E* − 10	1.06*E* − 11
60%	1.46*E* − 10	2.08*E* − 12	7.50*E* − 10	1.07*E* − 11
70%	1.47*E* − 10	2.10*E* − 12	7.56*E* − 10	1.08*E* − 11
80%	1.48*E* − 10	2.12*E* − 12	7.62*E* − 10	1.09*E* − 11
90%	1.49*E* − 10	2.13*E* − 12	7.69*E* − 10	1.10*E* − 11
100%	1.50*E* − 10	2.15*E* − 12	7.75*E* − 10	1.11*E* − 11
